# The Function of FK506-Binding Protein 13 in Protein Quality Control Protects Plasma Cells from Endoplasmic Reticulum Stress-Associated Apoptosis

**DOI:** 10.3389/fimmu.2017.00222

**Published:** 2017-03-02

**Authors:** Mini Jeong, Eunkyeong Jang, Suk San Choi, Changhoon Ji, Kyungho Lee, Jeehee Youn

**Affiliations:** ^1^Laboratory of Autoimmunology, Department of Anatomy and Cell Biology, College of Medicine, Hanyang University, Seoul, South Korea; ^2^Protein Metabolism Medical Research Center, Department of Biomedical Sciences, College of Medicine, Seoul National University, Seoul, South Korea; ^3^Department of Biological Sciences, Konkuk University, Seoul, South Korea

**Keywords:** plasma cells, long-lived plasma cells, FK506-binding protein 13, immunoglobulin, unfolded protein response, chaperone

## Abstract

Plasma cells (PCs) are exposed to intense endoplasmic reticulum (ER) stress imposed by enormous rates of immunoglobulin (Ig) synthesis and secretion. Therefore, protein homeostasis is crucial for the survival of PCs, but its molecular mechanism remains largely unknown. Here, we found marked overexpression of FK506-binding protein 13 (FKBP13) in long-lived PCs from autoimmune mice and investigated its function using a plasmacytoma cell line secreting IgA. FKBP13 expression was induced largely in the lumen of ER in response to treatment with an ER stressor tunicamycin or overexpression of an adaptive unfolded protein response (UPR) protein X-box binding protein 1 (XBP1). Silencing of FKBP13 expression led to induction of molecules involved in the terminal UPR and ER stress-associated apoptosis. FKBP13 interacted with Ig, facilitated its ubiquitination, and lowered the extent of ER stress. FKBP13 overexpression caused a significant reduction in secreted IgA in plasmacytoma cells, and FKBP13 knockdown exerted an opposite effect. Rapamycin interfered with the interaction between FKBP13 and IgA and enhanced the amount of secreted IgA. Importantly, the level of FKBP13 was inversely correlated with the amount of secreted antibody in long-lived PCs from autoimmune mice. These results suggest that FKBP13 is a marker of long-lived PCs and a component of XBP1-dependent ER protein homeostasis. FKBP13 is likely to act as a molecular chaperone that delivers misfolded ER clients, including Ig, to ER-associated degradation, so reducing proteotoxic stress on the PC. Our data reveal a novel cytoprotective role for FKBP13 in long-lived PCs occurring at the expense of antibody production.

## Introduction

Plasma cells (PCs) are the terminal effector cells of B lymphocytes, able to secrete copious amounts of antibodies [i.e., immunoglobulin (Ig) molecules], and thus constituting the humoral arm of adaptive immunity. PCs mostly die within 1 week in the secondary lymphoid organs where they develop. However, when PCs reside in milieu providing survival niches for them, such as bone marrow (BM) and inflamed sites, they terminally differentiate into post-mitotic PCs attaining a life span of months or even decades (1). These long-lived PCs have been known to be crucial for long-lasting immunity in experimental animals and humans (2–6), but little is known about how they sustain viability for such a long period.

Owing to their enormous rates of Ig synthesis and secretion, PCs are exposed to intense stress imposed by the assembly and trafficking of the abundant Ig cargo through the endoplasmic reticulum (ER). ER stress readily activates the adaptive strategy known as the unfolded protein response (UPR) to sustain viability while restoring protein homeostasis (7). However, when the ER stress becomes excessive and so beyond the capacity of adaptive UPR, the terminal UPR is instead triggered and executes the apoptotic program. The expression of the proapoptotic factor CHOP is a characteristic marker for terminal UPR leading to apoptosis (8). Several reports demonstrating the interplay between increased secretory load and ER stress-associated apoptosis in PCs suggest that long-lived PCs may be less sensitive to the latter (9, 10). However, the mechanism underlying how long-lived PCs evade ER stress-associated apoptosis remains largely unknown.

The UPR has three branches. Among these, the pathway mediated by inositol-requiring enzyme 1 and its downstream player, X-box binding protein 1 (XBP1), is known to participate in PC differentiation (11, 12). In the absence of XBP1, B cells develop normally to the mature state but yield PCs that secrete reduced amounts of Igs (13). XBP1 predominantly functions to promote the expansion of the ER and so allow PCs to secrete copious levels of Igs (14). These effects largely depend on its activity as a transcription factor. The transcriptionally induced target genes of XBP1 produce molecular chaperones to enhance the capacity of protein folding and ER-associated degradation (ERAD). During ERAD, molecular chaperones protect unfolded or misfolded proteins from spontaneous aggregation, facilitate their recognition by ubiquitin ligases, and finally deliver the ubiquitinated substrates to the proteasome (15). Thus, chaperones play a key role in the clearance of terminally misfolded proteins by the ubiquitin–proteasome system (UPS). Although several chaperone molecules involved in the folding and assembly of Ig chains, such as BiP/GRP78 and GRP94, have been characterized in B cells and PCs (16, 17), those important for protein homeostasis in long-lived PCs remain largely unexplored.

FK506-binding proteins (FKBPs) are a family of enzymes with peptidyl-prolyl *cis-trans* isomerase (PPIase, also known as rotamase) activity (18). In addition to this enzymatic activity, the PPIase domain contains a hydrophobic core that forms a drug-binding pocket, which allows FKBP to serve as an immunophilin. Among 15 mammalian FKBPs known to date, the prototypical member FKBP12 is the only one that has been shown to form complex with FK506 and rapamycin in the cytosol and mediate their immunosuppressive effects in T cells (19, 20). FK506–FKBP12 and rapamycin–FKBP12 complexes specifically inhibit calcineurin and mammalian target of rapamycin (mTOR), respectively. FK506-binding protein 13 (FKBP13) shares with FKBP12 approximately 43 and 51% homology at the levels of nucleotide and amino acid sequence, respectively (21). The conserved amino acid residues that comprise the drug-binding site of FKBP12 are completely conserved in FKBP13 (21). Nevertheless, the FK506–FKBP13 complex did not significantly inhibit calcineurin *in vitro* (22), and no function of a rapamycin–FKBP13 complex in a cell has been reported to data. It has been shown that FKBP13 is located in the lumen of the ER in canine pancreatic cells and is induced by ER stressors in Madin–Darby canine kidney cells (23, 24). However, whether FKBP13 plays an important role in PCs remains unknown to date.

Here, we investigated the role of FKBP13 in the UPR, apoptosis, and Ig production through the ER in PCs. We show that FKBP13 are more abundant in the ER of long-lived PCs compared to short-lived PCs and plays an essential role in the quality control of Ig in the ER. This proteostatic mechanism may contribute to the sustained survival of long-lived PCs at the expanse of secretory Ig production.

## Materials and Methods

### Plasmids and Reagents

pcDNA3.1, pcDNA-sXBP1 (25), pcDNA-CHOP (26), pGL3b-UPRE (carrying five copies of the UPRE domains) (27), and pRL-CMV (Promega) were used. Mouse FKBP13 cDNA was reverse-transcribed from RNA of RAW264.7 cells and inserted into MigR1 vector with myc tagging sequences (MigR1-myc-FKBP13). Plasmids carrying DNA sequences encoding shRNA specific for FKBP13 (pGFP-V-RS-shFKBP13) or scrambled shRNA (pGFP-V-RS-SCR) were purchased from Origene. Rapamycin, LPS, and PMA were obtained from Sigma-Aldrich and MG-132 from Millipore.

### Mice and Flow Cytometry

All animal experiments were performed in strict accordance with the recommendations in the Guide for the Animal Experimentation Ethics Committee of Hanyang University. The protocol was approved by the Institutional Animal Care and Use Committee of Hanyang University (permit numbers: HY-IACUC-16-0039 and HY-IACUC-16-0042). All methods were carried out in accordance with the guidelines and regulations.

NZB and NZW mice purchased from the Jackson Laboratory were crossed in a specific pathogen-free barrier facility at Hanyang University to obtain NZB/W F1 mice. KRN TCR transgenic mice on a C57BL/6 background (28) originally donated by Dr. Diane Mathis (Harvard Medical School, Boston, MA, USA) were kept in our animal facility and crossed with non-obese diabetic (NOD) and scurfy (*Foxp3^sf^*) mice to generate K/BxN and K/BxNsf strains, as described previously (29). Sanroque (*Roquin^san/san^*) mice (30) were purchased from Mutant Mouse Regional Resource Center and bred in our animal facility.

NZB/W F1 (36- to 40-week-old females), K/BxN (10-week-old males), K/BxNsf (4-week-old males), and sanroque (19- to 23-week-old) mice were fed drinking water containing 0.8 mg/ml bromodeoxyuridine (BrdU) (Sigma-Aldrich) for 14 days and assayed as described previously (29). In some experiments, sanroque mice were injected intraperitoneally with rapamycin dissolved in 1% ethanol and resuspended in 0.2% carboxymethylcellulose (Sigma-Aldrich) at a dose of 10 mg/kg of body weight/injection every 2 days for 14 days.

Fluorochrome-conjugated monoclonal antibodies to B220, CD138, BrdU, and IgG_1_ were purchased from eBiosciences or BD Biosciences. Anti-FKBP13 antibody (Santa Cruz) was conjugated with phycoerythrin (PE), according to the manufacturer’s instructions (Solulink). In brief, succinimidyl-4-formylbenzamide linker was conjugated with R-PE to produce preactivated, highly fluorescent R-PE. The antibody was incubated with succinimidyl-6-hydrazino-nicotinamide linker and then with preactivated R-PE in the presence of the TurboLink catalyst, leading to conversion of the antibody to conjugate through formation of stable bis-arylhydrazone bonds.

### Cell Culture and Transfection

J558 and BCL1 strains were purchased from ATCC, and M12.4.1 cells (31) were provided by Dr. Mark Boothby (Vanderbilt University, Nashville, TN, USA). J558 cells were cultured with DMEM (Welgene, Daegu, South Korea) supplemented with 10% horse serum (Welgene) and penicillin/streptomycin (Welgene). One million cells were transfected with 5 μg DNA by electroporation using Neon transfection system (Thermo Fisher Scientific) or NEPA21 Super Electroporator (Nepa Gene). Transfection efficiency was about 10–20%. Sixteen hours after transfection, the cells were harvested and subjected to further experiments. Cell viability was measured by the trypan blue exclusion method. To detect cell death, cells were stained with 7-aminoactinomycin D (7-AAD) and Annexin V (BD Biosciences) and assayed by flow cytometry.

### Immunocytochemistry

J558 cells were treated with 10 μg/ml tunicamycin (Tm) or DMSO for 48 h. The cells were plated on coverslips coated with poly-l-lysine (Sigma-Aldrich) for 1 h, fixed with 4% paraformaldehyde in PBS for 15 min, and permeabilized with 0.5% Triton X-100 in PBS for 15 min. After blocking with 2% bovine serum albumin in PBS for 1 h, the cells were incubated with a mixture of goat anti-FKBP13 (Santa Cruz) and mouse anti-KDEL antibodies (Abcam) for 2 h and then with a mixture of anti-goat IgG conjugated with Alexa Fluor 555 and anti-mouse IgG conjugated with Alexa Fluor 488 (Thermo Fisher Scientific). Cells were washed with PBS three times between steps. The coverslips were mounted on slides using VECTASHIELD Hardset Antifade Mounting Medium with DAPI (Vectorlabs). Fluorescence images were visualized using a confocal microscope (Zeiss LSM 700) and analyzed using ZEN 2012 SP2 (blue edition, ver.1.1.2.0).

### Immunoprecipitation and Western Blot Analysis

Cells were incubated with RIPA buffer (GenDEPOT) containing a mixture of protease inhibitors and phosSTOP (all from Roche) on ice and spun down to obtain lysates. Forty μg aliquots of lysates were assayed by SDS-PAGE and western blotting. Proteins were electrotransferred to Immobilon-P membranes (Millipore). For immunoprecipitation, cells were lysed with Xpro IP cell lysis buffer (GenDEPOT) containing a mixture of protease inhibitors (Roche). One mg of lysate was incubated with 1 μg of primary antibody or normal goat IgG overnight at 4°C, and then another overnight after adding 10 μg of protein G-agarose beads (Santa Cruz). Beads were washed and assayed by SDS-PAGE and western blotting. Anti-caspase-3 and anti-ubiquitin antibodies were purchased from Cell Signaling. Anti-FKBP13, anti-PARP1/2, anti-β-actin, and anti-GAPDH antibodies were obtained from Santa Cruz and anti-mouse IgA antibody from Southern Biotech. In some experiments, cells were pretreated with 100 nM rapamycin for 24 h before lysis.

### Quantitative RT-PCR

Total RNA was purified using Qiazol reagent (Qizgen). cDNA was synthesized using amfiRivert cDNA Synthesis Master Mix (GenDEPOT) and amplified by quantitative PCR using SYBR Green Master Mix (Thermo Fisher Scientific). Relative amounts of each gene transcript were normalized to the amounts of β2 microglobulin or GAPDH transcripts. Primer sequences used were as follows: FKBP11 (GGTCATAGAACTCGGCCAAA and CAGCTCCACATCGTACTGCA), FKBP13 (TGACAGCAGCCTACCACAGA and AGCTCCACCTCAAACACCAG), spliced XBP1 (sXBP1) (GAGTCCGCAGCAGGTG and GTGTCAGAGTCCATGGGA), XBP1 (AAGAACACGCTTGGGAATGG and ACTCCCCTTGGCCTCCAC), ATF4 (AACCTCATGGGTTCTCCAGCGA and CTCCAACATCCAATCTGTCCCG), CHOP (TGAAATTGGGGGCACCTATA and CTGCTCCTTCTCCTTCATGC), Cα (TAACATGGACCAAGGTGCCC and GTTTGCTCCAGTAGCTGGGT), EDEM1 (GCTGCGTATCAGAGCATCCAGA and CAGCGAGTCAATCCAGGTGTTC), EDEM2 (GACCCTGTGTTTGAAGATGTGGC and CACTTGCCAGTGAGCACATCGA), Derl3 (GCAACTCGGTTGTCACAGACCT and CTGAGGGTCATCTAGTAGCAGC), Syvn1 (CCAACATCTCCTGGCTCTTCCA and CAGGATGCTGTGATAAGCGTGG), PDI (GTGTTGGAACTGACGGACGA and GGCAGTGCAATCCACCTTTG), BiP (CATGGTTCTCACTAAAATGAAGGA and GCTGGTACAGTAACAACTG), GRP94 (GTTTCCCGTGAGACTCTTCAGC and ATTCGTGCCGAACTCCTTCCAG), Blimp1 (ACCGTCTTGAGGACATGGAG and GTTGCTGTGAGGCAACTTCA), β2 microglobulin (TGACCGGCCTGTATGCTATC and CAGTGTGAGCCAGGATATAG), and GAPDH (CATCACTGCCACCCAGAAGACTG and ATGCCAGTGAGCTTCCCGTTCAG).

### Enzyme-Linked Immunosorbent Assays (ELISA)

Sixteen hours after transfection of J558 cells with MigR1, MigR1-myc-FKBP13, pGFP-V-RS, or pGFP-V-RS-shFKBP13, GFP-positive cells were sorted using a FACS Aria III (BD Biosciences) and cultured in DMEM with 10% horse serum. After 48 h, supernatants were collected and assayed by sandwich ELISA to measure secreted IgA. In brief, immunosorbent plates (Thermo Fisher Scientific) precoated with 1.2 μg/ml goat anti-mouse IgA were incubated with the spent media or serially diluted mouse IgA followed by anti-mouse IgA-HRP. All antibodies were purchased from Southern Biotech.

### Luciferase Assay

Cells transfected with MigR1, MigR1-myc-FKBP13, pGL4b, pGL3b-UPRE, and/or pRL-CMV were assayed with a Dual-luciferase assay system (Promega). Firefly luciferase activity was normalized by *Renilla* luciferase activity and displayed as relative luciferase units.

### Statistical Analysis

Data are presented as means ± SEMs. Differences between groups were evaluated by unpaired Student’s *t*-tests. Also, *p* values are indicated when differences between two groups were statistically significant (<0.05).

## Results

### FKBP13 Is a Marker of Long-Lived PCs

**Figure 1:**
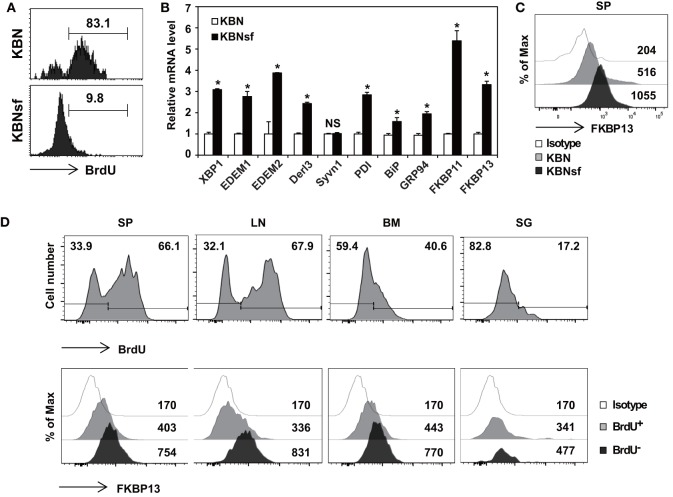
**Enhanced expression of FK506-binding protein 13 (FKBP13) in long-lived plasma cells**. **(A)** Splenocytes from K/BxN and K/BxNsf mice fed with bromodeoxyuridine (BrdU)-containing water for 14 days were examined by BrdU incorporation assays. FACS profiles gated on B220^lo^CD138^+^ cells are shown. **(B)** B220^lo^CD138^+^ cells were sorted from splenocytes and assayed by quantitative RT-PCR. **(C)** Splenocytes were stained with anti-FKBP13-PE and assayed by FACS. The numbers indicate the mean fluorescence intensities of staining. **(D)** NZB/W F1 mice were *in vivo* labeled with BrdU as in **(A)**. Spleen (SP), lymph nodes (LN), bone marrow (BM), and salivary glands (SG) were removed, and single cell suspensions were prepared and assayed by FACS. The histograms in the upper panel show the intensity of BrdU staining with the percentages of BrdU^−^ and BrdU^+^ cells among the B220^lo^CD138^+^ cells. The histograms in the lower panel show the intensities of FKBP13 staining with the values of the mean fluorescence intensities of cells gated on BrdU^+^ and BrdU^−^ of the upper panel histograms. Isotype, isotype-matched control antibody staining. The data are representative of two **(A–C)** or four **(D)** independent experiments. *p < 0.05 by Student’s *t*-test. NS, not significant.

We have previously shown that the vast majority of splenic PCs are short-lived in autoimmune arthritic K/BxN mice and long-lived in K/BxNsf mice, the congenic strain carrying the scurfy allele (29). By using BrdU incorporation assays, we found that approximately 83% of the splenic PC populations in K/BxN mice were BrdU^+^ indicative of dividing, rapidly turning over cells, whereas approximately 90% of those in K/BxNsf mice were BrdU^−^ long-lived cells that survived for at least 14 days without cell division (Figure 1A). To determine whether the difference in the life span between short-lived PCs and long-lived PCs correlates to the folding capacity and UPR status of the ER, we sorted splenic PCs (B220^lo^CD138^+^) from K/BxN and K/BxNsf mice and compared the expression of genes encoding molecules involved in the XBP1 branch of the UPR. The level of *Xbp1* transcripts was higher in PCs from K/BxNsf mice than in those from K/BxN mice. Transcripts encoding molecules with protein-folding capacity, such as PDI, BiP, and GRP94 (16, 17, 32), were also more abundant in PCs from K/BxNsf mice. The difference was more prominent for PDI transcripts and marginal for BiP and GRP94 transcripts. Approximately 2.5- to 4-fold increases in K/BxNsf PCs were observed for ERAD components, such as EDEM1, EDEM2, and Derl3, but not Syvn (33). Interestingly, the transcripts of two poorly characterized FKBP proteins, FKBP11 and FKBP13, were also more abundant in K/BxNsf PCs (Figure 1B). The upregulation of FKBP13 in K/BxNsf PCs was confirmed at the protein level (Figure 1C).

To determine whether FKBP13 is a general marker of long-lived PCs, we carried out the same assays with F1 female hybrids of NZB and NZW strains (referred to hereafter as NZB/W F1), a canonical model of lupus and also known to have a substantial number of long-lived PCs in the spleen (SP) (34). Although the ratio of BrdU^+^ PCs to BrdU^−^ PCs varied with the organ, FKBP13 expression was invariably higher in BrdU^−^ PCs than in their BrdU^+^ counterparts, regardless of organ (Figure 1D). These results, together with the data from K/BxNsf SPs, demonstrate that the levels of FKBP13 and protein-folding capacity are upregulated when PCs become long-lived.

### FKBP13 Is a Component of XBP1-Dependent ER Protein Homeostasis in Long-Lived PCs

**Figure 2:**
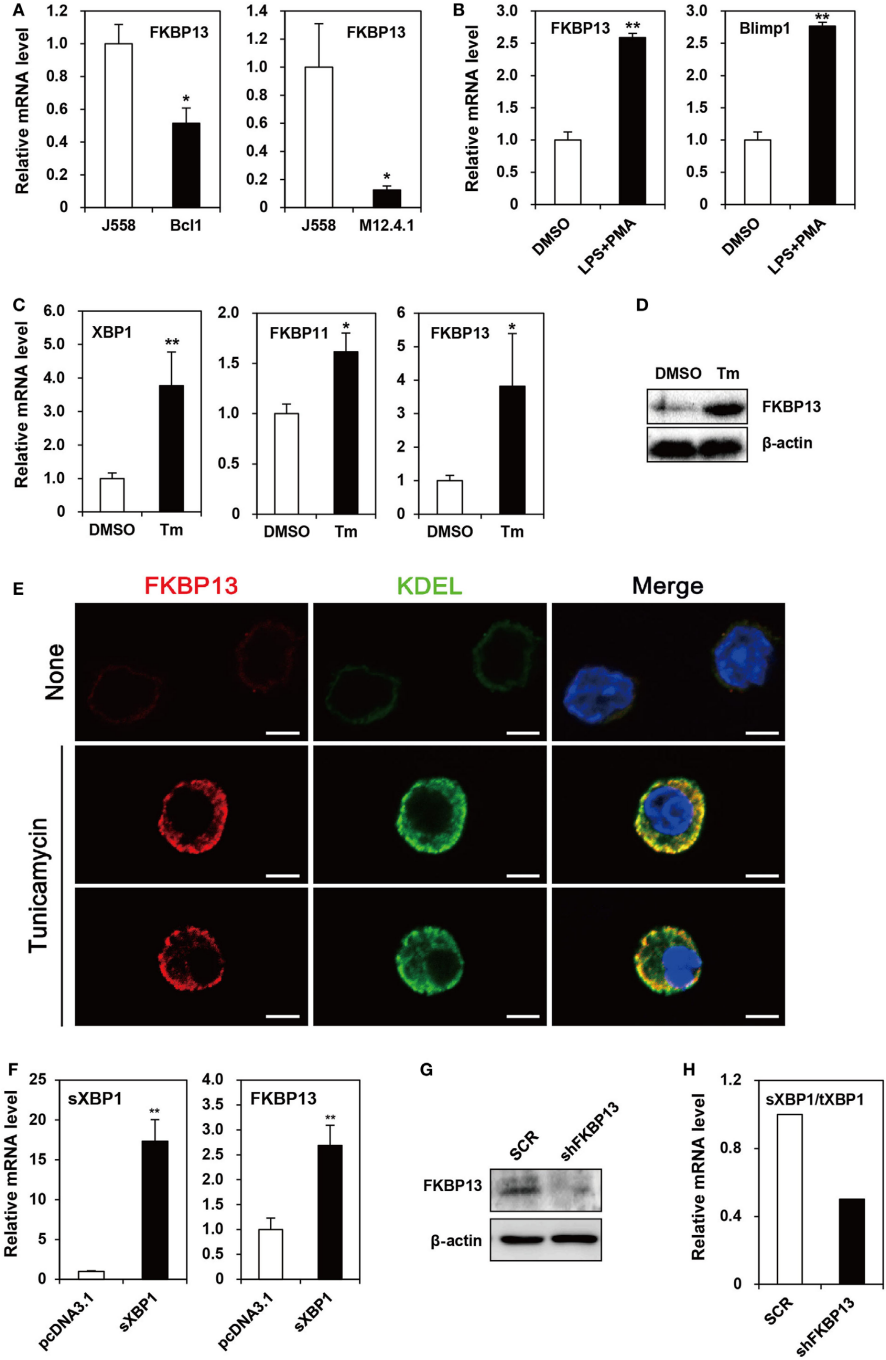
**FK506-binding protein 13 (FKBP13) is a component of the X-box binding protein 1 (XBP1) pathway of the unfolded protein response in plasmacytoma cells**. **(A)** J558, M12.4.1, and BCL1 cells were assayed by quantitative RT-PCR methods to compare levels of FKBP13 transcripts. **(B)** BCL1 cells were treated with 10 μg/ml LPS plus 5 ng/ml PMA for 3 h, and levels of FKBP13 and Blimp1 mRNA were analyzed by quantitative RT-PCR. **(C–E)** J558 cells were treated with 10 μg/ml tunicamycin (Tm) or vehicle DMSO for 48 h, followed by quantitative RT-PCR **(C)**, immunoblotting **(D)**, and fluorescence microscopy after staining for FKBP13 (red) and KDEL (green) **(E)**. Scale bars, 10 μm. (**F)** J558 cells were transfected with pcDNA3.1 or pcDNA-sXBP1 (sXBP1), incubated with 0.8 mg/ml G418 for 24 h and assayed by quantitative RT-PCR. **(G,H)** J558 cells were transfected with pGFP-V-RS-shFKBP13 (shFKBP13) or pGFP-V-RS-SCR (SCR) and assayed by immunoblotting **(G)** and quantitative RT-PCR **(H)**. sXBP1/tXBP1 indicates the ratio of spliced XBP1 to total XBP1. All data are representative of at least three independent experiments. *p < 0.05 and **p < 0.01 by Student’s *t*-test.

Because primary PCs mostly die spontaneously during *in vitro* culture, we needed a model cell line that mimics long-lived PCs to examine functional features of FKBP13 *in vitro*. To this end, we chose as a model cell line, the J558 murine plasmacytoma cell line, which at least partially mirrors the characteristics of long-lived PCs, such as secretion of monoclonal antibody and defects in apoptosis. J558 cells contained FKBP13 mRNA at a level higher than two lymphoma cell lines derived from murine mature B cells, M12.4.1 and BCL1 (Figure 2A). Interestingly, when BCL1 cells were stimulated with a mixture of LPS and PMA to induce the expression of Blimp1, the master regulator of PC differentiation, FKBP13 became more abundant (Figure 2B). This suggests that the expression of FKBP13 is induced during PC differentiation.

Since PCs require the XBP1-mediated pathway of UPR to maintain protein homeostasis, we tested whether FKBP13 is involved in this process using J558 cells. When the cells were treated with Tm to induce XBP1 expression, FKBP11 and FKBP13 transcripts were elevated (Figure 2C). The induction of FKBP13 was more prominent than that of FKBP11 and was confirmed at the protein level (Figure 2D).

To address whether the upregulation of FKBP13 in long-lived PCs is functionally related to the homeostasis in the ER, we determined the intracellular localization of FKBP13 in J558 cells. Although these cells in suspension culture were not an ideal model to analyze proteins’ intracellular distributions, immunostaining analysis followed by confocal microscopy showed that FKBP13 marks specific subcellular compartments (Figure 2E). The staining pattern of FKBP13 was strikingly similar to that obtained by an antibody to the KDEL sequence, which marks ER lumen-residing proteins. These results suggest that FKBP13 primarily localizes in the lumen of ER.

To address the functional relationship between XBP1 and FKBP13, J558 cells were transfected with cDNA encoding the spliced (i.e., active) form of XBP1 (sXBP1). The overexpression of sXBP1 markedly induced FKBP13, indicating that FKBP13 is a downstream target of XBP1 (Figure 2F). To further examine the function of FKBP13 in the ER homeostasis of long-lived PCs, we silenced FKBP13 mRNA by overexpression of specific shRNA (Figure 2G). FKBP13 silencing decreased the ratio of sXBP1 to total XBP1 (Figure 2H), suggesting that the expression of XBP1 and FKBP13 is tightly regulated by each other. These results suggest that FKBP13 is a component of XBP1-dependent protein homeostasis in the ER of plasmacytoma cells.

### FKBP13 Silencing Induces ER Stress-Associated Apoptosis in Plasmacytoma Cells

**Figure 3:**
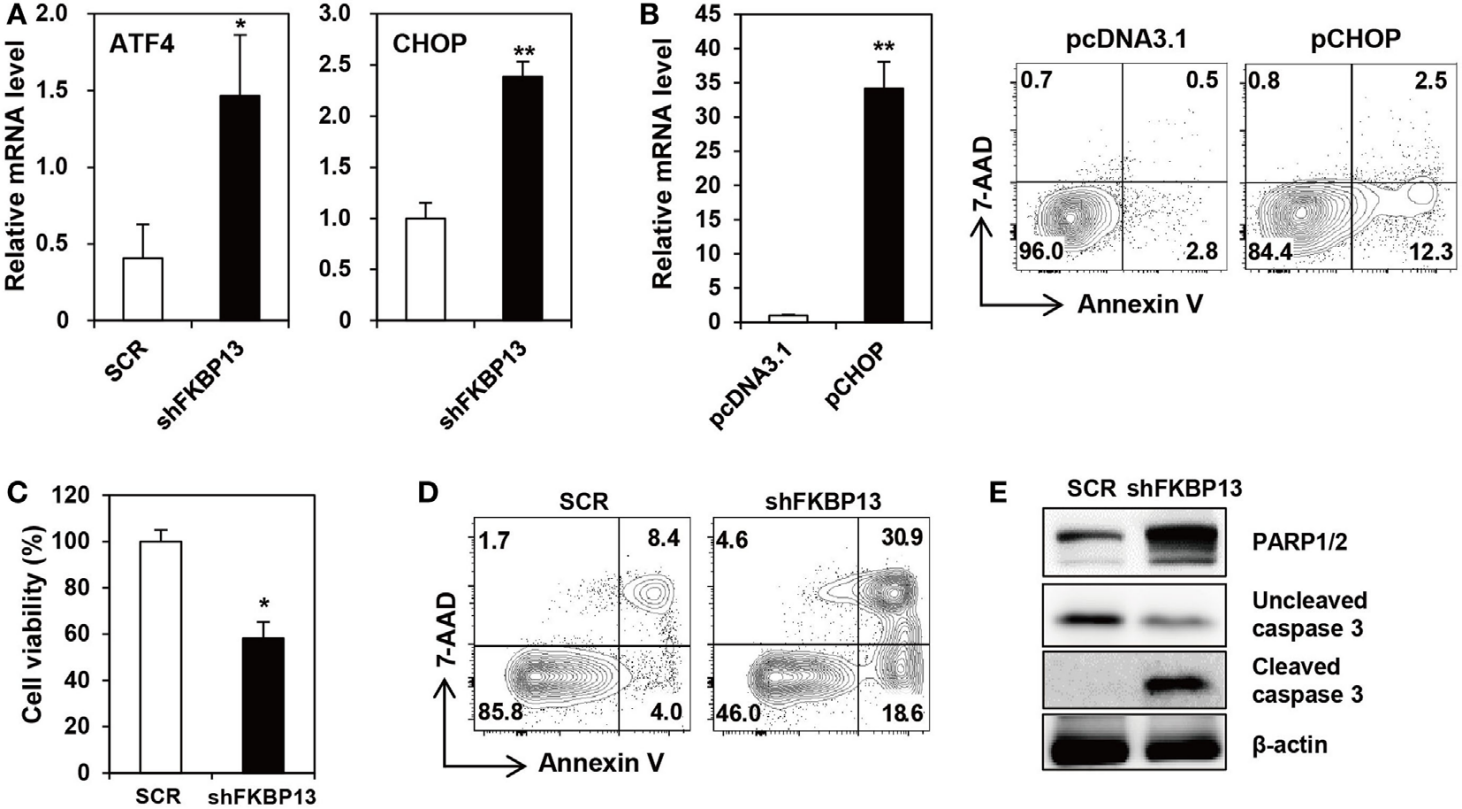
**FK506-binding protein 13 (FKBP13) silencing induces apoptotic death of plasmacytoma cells**. **(A,C–E)** J558 cells were transfected with pGFP-V-RS-shFKBP13 (shFKBP13) or pGFP-V-RS-SCR (SCR). The cells were harvested 16 h after transfection and assayed by quantitative RT-PCR **(A)**, trypan blue exclusion **(C)**, FACS after staining with Annexin V and 7-aminoactinomycin D (7-AAD) **(D)**, and immunoblotting **(E)**. **(B)** J558 cells were transfected with pcDNA3.1 or pcDNA-CHOP (pCHOP) and assayed by quantitative RT-PCR for CHOP expression (left) and by FACS after staining with Annexin V and 7-AAD (right). All data are representative of more than three independent experiments. *p < 0.05 and **p < 0.01 by Student’s *t*-test.

Various cellular stresses cause the accumulation of misfolded proteins in the ER, triggering the UPR (7). During the early phase, the UPR increases the synthesis of folding factors to enhance protein-folding capacity and halts protein synthesis to reduce ER loading. However, if proteasomal inhibition prolongs, the UPR triggers so-called “ER stress-associated cell death” (35). To determine whether FKBP13 plays a role in ER stress-associated cell death in long-lived PCs, we silenced FKBP13 mRNA and examined the expression of UPS-associated proapoptotic transcription factors, ATF4, and its downstream molecule CHOP. FKBP13 knockdown markedly induced the expression of ATF4 and CHOP (Figure 3A). To confirm that CHOP is induced in the course of apoptosis, we overexpressed CHOP and monitored Annexin V^+^ cells using cell sorting analysis. The overexpression of CHOP markedly increased the frequencies of Annexin V^+^ 7-AAD^−^ early apoptotic and Annexin V^+^ 7-AAD^+^ late apoptotic cells (Figure 3B). Consistently, trypan blue exclusion assay showed that FKBP13 knockdown resulted in increased cell death in J558 plasmacytoma cells (Figure 3C), which correlated to upregulation of apoptotic markers, such as Annexin V positivity, caspase-3 cleavage, and PARP1/2 expression (Figures 3D,E). These results demonstrate that FKBP13 loss readily triggers ER stress-associated apoptosis in PCs.

### FKBP13 Has a Role in Protein Quality Control in the ER of Plasmacytoma Cells

**Figure 4:**
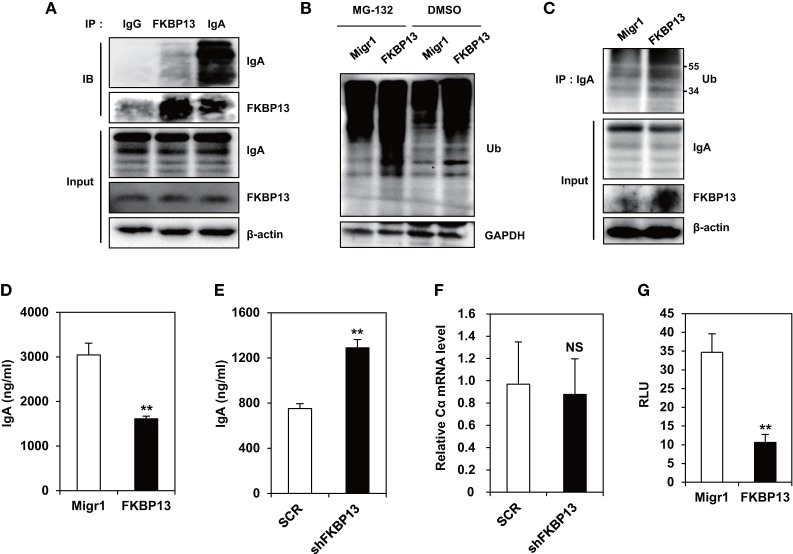
**FK506-binding protein 13 (FKBP13) promotes ubiquitination-mediated degradation of immunoglobin (Ig) molecules**. **(A)** J558 cells were lysed, immunoprecipitated with anti-goat IgG, anti-FKBP13, or anti-IgA antibodies, and assayed by western blotting. **(B–G)** J558 cells were transfected with MigR1, MigR1-myc-FKBP13 (FKBP13), pGFP-V-RS-shFKBP13 (shFKBP13), or pGFP-V-RS-SCR (SCR) as indicated. **(B)** The transfectants were incubated with 1 μM MG-132 or DMSO for 24 h followed by western blotting. **(C)** Lysates from the transfectants were immnoprecipitated with anti-IgA antibodies and assayed by western blotting. **(D,E)** GFP^+^ transfectants were sorted by FACS and cultured for 24 h, and supernatant IgA was assayed by enzyme-linked immunosorbent assays. **(F)** IgA Cα transcripts in the transfectants were assayed by quantitative RT-PCR. **(G)** J558 cells were transfected with MigR1 or MigR1-myc-FKBP13 constructs together with reporter constructs (pGL3b-UPRE and pRL-CMV), and dual luciferase activities were measured. Firefly luciferase activity was normalized by the *Renilla* luciferase activity and is shown as relative luciferase units (RLU). All data are representative of at least three independent experiments. *p < 0.05 and **p < 0.01 by Student’s *t*-test. NS, not significant.

To determine the role of FKBP13 in quality control of Ig, we performed coimmunoprecipitation assay between FKBP13 and Ig in J558 cells. We found that FKBP13 physically interacted with IgA molecules (Figure 4A), suggesting that FKBP13 acts as a molecular chaperone for incoming ER clients, such as Ig nascent polypeptides. FKBP13 was coimmunoprecipitated with goat IgG used as a negative control antibody, albeit to a much less extent than with anti-mouse IgA antibody (the first lane of Figure 4A), suggesting interaction between free FKBP13 and goat IgG *in vitro*. During the folding cycle in the ER lumen, terminally misfolded proteins are collected by molecular chaperones and delivered to the ERAD, in which the substrates are ubiquitinated by E3 ubiquitin ligases and degraded by the proteasome (15, 16). We therefore asked whether FKBP13 is required for protein quality control in the ER. Notably, the overexpression of FKBP13 resulted in the accumulation of ubiquitin-conjugated protein species (Figure 4B). Such a trend was similarly observed when J588 cells were treated with the proteasome inhibitor MG132. Interestingly, the accumulated ubiquitin conjugates included IgA (Figure 4C). These results suggest that FKBP13 participates in quality control of ER clients, including Ig, possibly as a molecular chaperone that delivers terminally misfolded proteins to ERAD.

### FKBP13 Regulates the Secretion of Ig and Counteracts ER Stress

Ubiquitination and degradation of Igs influence the amounts of secreted Ig molecules. To determine whether FKBP13 influences the secretion of functional Igs, we overexpressed FKBP13 in J588 cells and measured the amount of secreted IgA. FKBP13 overexpression caused a significant reduction in secreted IgA (Figure 4D). Likewise, FKBP13 knockdown exerted an opposite effect, resulting in an increased amount of secreted IgA (Figure 4E). This effect is likely to take place at the post-transcriptional level, because FKBP13 silencing did not alter the mRNA level of IgA heavy chain (Figure 4F).

Ubiquitination and degradation of surplus Igs reduce ER stress. To determine whether FKBP13 counteracts ER stress, we performed UPRE-luciferase assays using an XBP-1-dependent reporter construct. As shown in Figure 4G, FKBP13 overexpression dramatically lowered the XBP1-driven luciferase expression, i.e., the level of ER stress. This result indicates that FKBP13 counteracts ER stress. Our results, taken together, suggest that FKBP13 acts as a chaperone targeting Ig molecules to the ubiquitin-mediated degradation pathway. This proteostatic mechanism, functioning at the expense of Ig secretion, lowers ER stress to a level that does not trigger terminal UPR-mediated death.

### Rapamycin Inhibits the Activity of FKBP13 in Plasmacytoma Cells and Depletes Long-Lived PCs in Autoimmune Mice

**Figure 5:**
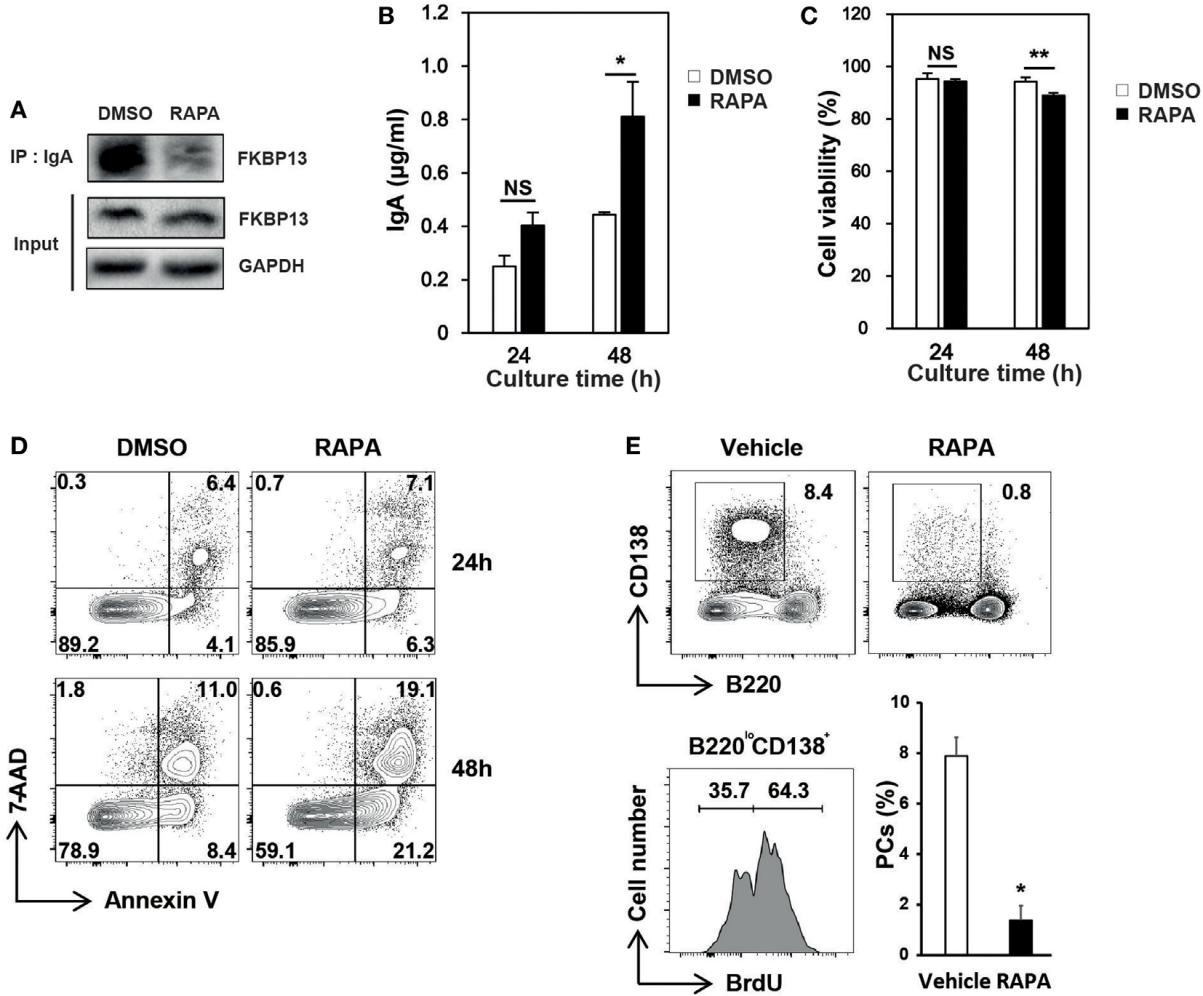
**Effects of rapamycin on the functioning of FK506-binding protein 13 (FKBP13)**. J558 cells were cultured in the presence or absence of 100 nM rapamycin. **(A)** After 24 h, cells were assayed by immunoprecipitation methods. **(B)** After 24 or 48 h, the culture supernatants were assayed by enzyme-linked immunosorbent assays to determine the amounts of IgA secreted by 10^5^ viable cells. **(C)** Cell viability was determined by trypan blue exclusion. **(D)** Cells were stained with 7-aminoactinomycin D (7-AAD) and Annexin V, followed by FACS analyses. All data are representative of three independent experiments. **(E)** Sanroque mice were fed with bromodeoxyuridine (BrdU)-containing water and injected with rapamycin for 2 weeks, followed by FACS analysis. Representative FACS profiles with percentages of cells within indicated areas and mean ± SEM (*n* = 2–3 mice/group) are shown. *p < 0.05 and **p < 0.01 by Student’s *t*-test. NS, not significant.

Molecular chaperones recognize a stretch of hydrophobic amino acids exposed on the clients’ surface (15, 16). The PPIase domain of FKBP13 contains a hydrophobic core that forms a drug-binding pocket. We speculated that the binding of rapamycin to FKBP13 may interfere with FKBP13 interaction with Ig molecules and, thus, may inhibit the activity of FKBP13 as a molecular chaperone. To test this idea, we treated J558 cells with rapamycin for 24–48 h and immunoprecipitated IgA. Immunoblotting analysis showed that rapamycin reduced the amount of FKBP13 which is coimmunoprecipitated with IgA (Figure 5A). This result is consistent with the possibility that rapamycin blocks the hydrophobic core of the PPIase domain that also binds misfolded/unfolded amino acids of IgA. Next, we reasoned that if rapamycin indeed binds FKBP13 and inhibits its chaperone activity, rapamycin would enhance Ig production. Indeed, rapamycin-treated cells secreted more IgA as compared with untreated cells (Figure 5B). Although rapamycin was somewhat cytotoxic, inducing apoptosis (Figures 5C,D), these results together support our hypothesis that FKBP13 acts as a molecular chaperone for Igs in the ER lumen of PCs.

The sanroque mouse strain is a lupus model, in which hyperactivation of follicular helper T cells precipitates autoantibody production and lupus nephritis (30). We found that, like NZB/W F1 mice, sanroque mice exhibit accumulation of long-lived PCs in their SP and lymph nodes (LN); approximately 36% of PC (B220^lo^CD138^+^) population was BrdU^−^ in BrdU incorporation assay (Figure 5E). To determine whether the effect of rapamycin is sufficient to deplete long-lived PCs *in vivo*, we treated sanroque mice with rapamycin for 2 weeks and measured remaining PCs. Treatment with rapamycin dramatically reduced the proportion of PCs by approximately 83%, suggesting a potential of rapamycin to deplete long-lived PCs.

### Antibody Secretion Is Compromised in Long-Lived PCs

**Figure 6:**
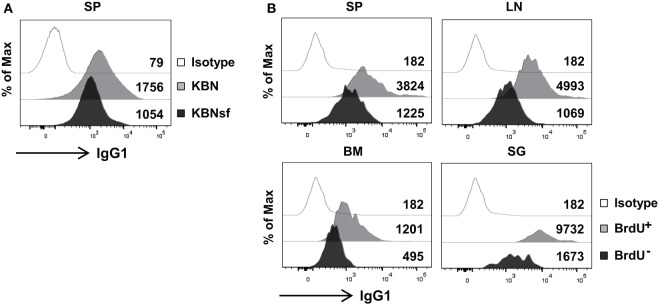
**Compromised immunoglobin (Ig) production in long-lived plasma cells**. **(A)** Splenocytes from K/BxN and K/BxNsf mice were stained for B220, CD138, and intracellular IgG_1_, followed by FACS analysis. Histograms gated on B220^lo^CD138^+^ cells with mean fluorescence intensities are shown. **(B)** Spleen (SP), lymph nodes (LN), bone marrow (BM), and salivary glands (SG) were removed from NZB/W F1 mice that had been labeled with BrdU for 14 days. The single cell suspensions extracted from the organs were assayed by FACS. Histograms gated on B220^lo^CD138^+^BrdU^+^ or B220^lo^CD138^+^BrdU^−^ are shown along with mean fluorescence intensities. Isotype, isotype-matched control antibody staining. Data are representative of two **(A)** or four **(B)** independent experiments.

The aforementioned data obtained from plasmacytoma cells suggest that FKBP13 is involved in the balance between Ig production and the viability/longevity of PCs. We evaluated whether this trade-off exists in primary PCs. Splenic PCs from K/BxNsf mice, which were mostly long-lived and FKBP13^hi^, had lower levels of intracellular IgG_1_ than those from K/BxN mice, which were mostly short-lived and FKBP13^lo^ (Figure 6A). In agreement with this, in NZB/W F1 mice labeled with BrdU for 2 weeks, BrdU^−^ PCs in SPs, LN, BM, and salivary glands contained lower levels of intracellular IgG_1_ than BrdU^+^ PCs (Figure 6B). These results suggest that FKBP13 is involved in the trade-off between viability/longevity and antibody production in long-lived PCs.

## Discussion

We have described here a novel function of FKBP13 in PCs that is distinct from their PPIase and immunophilin activities. Our study indicates that, as an XBP1-driven UPR protein, FKBP13 chaperones surplus Ig molecules to the ubiquitin-mediated degradation system. As a result, PCs relieve ER stress and evade terminal UPR-mediated cell death. At the same time, the production of secretory antibodies is inevitably compromised. Thus, our results suggest that FKBP13 has a key role in the trade-off between viability and antibody production in long-lived PCs.

How FKBP13 recognizes aberrant proteins remains to be elucidated. However, we hypothesize that the hydrophobic, drug-binding domain of FKBP13 selectively interacts with hydrophobic patches of unfolded proteins including nascent Igs that are not exposed in the native protein confirmation. Our finding that rapamycin interferes with the interaction between FKBP13 and Ig molecules strongly supports this hypothesis. By recognizing such aberrant sequences, FKBP13 may be able to deliver such protein cargos to the ubiquitin ligation system. In so doing, FKBP13 may form a transient network involving other chaperones and co-chaperones to orchestrate the complex tasks required. The detailed mechanisms underlying this process are currently under investigation.

Our data indicating the possibility of rapamycin–FKBP13 binding is interesting since previous studies have exclusively pointed to cytosolic FKBP12 as a rapamycin-binding partner (20, 36). The rapamycin–FKBP12 complex is known to inhibit the mTOR pathway by directly binding to the mTOR complex 1 (37). We speculate that, just as the FK506–FKBP13 complex does not inhibit calcineurin (22), the rapamycin–FKBP13 complex does not directly inhibit the mTOR pathway, because its localization is restricted to the lumen of ER. Instead, rapamycin may deprive FKBP13 of the possibility to recognize and chaperone nascent Ig proteins. If this is the case, PCs may respond to rapamycin by two separate pathways—rapamycin–FKBP12–mTOR and rapamycin–FKBP13–UPS axes—that finally converge on the pathway to cell death. Our data do not indicate which pathway contributes more to the cytotoxic effect of rapamycin in plasmacytoma cells. However, we noted with interest that expression of the two FKBPs is regulated in opposite directions by ER stress: FKBP12 expression was decreased and FKBP13 expression increased, in response to Tm (data not shown). In this regard, the action of rapamycin on FKBP13 chaperone function may be more important for perturbing the physiology of long-lived PCs under hyper-ER stress.

Although it is clear that FKBP13 expression is inversely correlated with the level of Ig secretion in PCs, the reduced antibody production seen in long-lived PCs is not necessarily due to FKBP13-mediated stimulation of ERAD, because PCs operate systems other than UPR, such as autophagy, for maintaining protein homeostasis (38). Autophagy engulfs cytoplasmic contents and breaks down protein aggregates in double-membraned vesicles that are delivered to lysosome. Autophagy, if it occurs, may have a similar outcome to FKBP13-mediated ERAD, in terms of limiting Ig synthesis while sustaining viability. Indeed, the ERAD system is functionally connected to autophagy, since the ubiquitin system and chaperones are known to trigger autophagy (39). It would be worth seeing whether autophagy is more active in long-lived PCs than short-lived PCs in our system and whether FKBP13 can promote the initiation of autophagy.

We acknowledge that the cytotoxic effect of FKBP13 silencing was significant but not sufficient to deplete all plasmacytoma cells, perhaps due in part to the low efficiency of DNA delivery into plasmacytoma cells (usually 10–20%), which may lead to underestimating the effectiveness of FKBP13. It is also possible that other chaperones partially complement a deficiency of FKBP13. We suspect that FKBP11 can substitute for FKBP13, given that the PPIase domains of these two ER-resident proteins have 46% amino acid identity (22). Our data showing upregulation of BiP and GPR94 in long-lived PCs, albeit to modest extents, raise the question of whether these proteins cooperate with FKBP13 in targeting surplus Ig proteins to proteasomal degradation in long-lived PCs, in addition to their roles in the folding and assembly of Ig chains (40). Nevertheless, we have clearly demonstrated here that FKBP13 has a specific function in PCs not completely complemented by other chaperones.

The sustained presence of autoreactive long-lived PCs in peripheral lymphoid organs has the potential to exacerbate pathologic effects by several mechanisms. First, in addition to their continuous production of antibodies, these cells are refractory to immunosuppressive drugs, including steroids, cyclophosphamide, and rituximab (2, 3, 34). Second, they can become more active after B cell depletion therapy in some patients with autoimmune diseases (4, 5). Third, they evade immune complex-mediated cell death by downregulating the death receptor FcγRIIb (29). Finally, they promote the differentiation of follicular helper T cells, so forming a positive feedback loop that amplifies humoral immunity (6). Therefore, a strategy limiting the longevity of these cells would be beneficial in controlling diseases related to long-lived PCs and plasmacytoma cells. Our data indicate that FKBP13 could be a novel therapeutic target for depleting pathogenic long-lived PCs.

In conclusion, we have identified FKBP13 as a key regulator of protein homeostasis in long-lived PCs. FKBP13 confers survival on PCs at the expense of antibody secretion. This insight into the mechanisms that control the viability of long-lived PCs and malignant PCs could lead to new strategies for treating autoimmunity and myeloma.

## Author Contributions

MJ, EJ, SC, and CJ performed experiments and prepared figures. MJ and JY designed experiments and interpreted data. KL provided analysis tools and interpreted data. MJ, SC, and JY wrote the manuscript. JY supervised the study. All the authors reviewed the manuscript.

## Conflict of Interest Statement

The authors declare that the research was conducted in the absence of any commercial or financial relationships that could be construed as a potential conflict of interest.
